# Supplementary material for: Exploring the genetic basis of human population differences in DNA methylation and their causal impact on immune gene regulation
Source: Genome Biol. 2018 Dec 18;19:222. doi: 10.1186/s13059-018-1601-3 (PMC6299574; doi:10.1186/s13059-018-1601-3)
Supplement: Supplementary file 1 — Figure S1. Overview of the EvoImmunoPop experimental setting. Figure S2. Exploring the non-linear effects of age on DNA methylation. Figure S3. Mono-DMS were detected using the same approach as described in the Materials and methods section, and including the proportions in monocyte subpopulations as covariates. Figure S4. PCA of the genetic data, based on 151,419 SNPs, for Africans (AFB, red dots) and Europeans (EUB, blue dots). Figure S5. Fine mapping of meQTLs. Figure S6. Histogram of physical proximity of cis-meQTLs. Figure S7. Genomic location of CpG sites associated with a meQTL. Figure S8. Proportions of population differences in DNA methylation accounted for by genetics. Figure S9. Fold enrichment of meQTLs in GWAS hits. Figure S10. Fold enrichment of meQTLs associated with DMS in GWAS hits related to “immune system disorder”. Figure S11. Rationale for the detection of trios to be used for causality inference. Figure S12. Cartoons of the various simulated scenarios. Figure S13. Heat map of correlation between the first ten PCs of expression and DNA methylation. Figure S14. Genomic location of eQTMs (NS) and reQTMs (for all stimulated conditions). Figure S15. Number of reQTM-genes, per condition, according to the direction of their association with DNA methylation. Figure S16. Causality inference upon immune stimulation. (PDF 3088 kb) [file 13059_2018_1601_MOESM1_ESM.pdf]

Additional file 1

Supplementary Figures S1-S16

Exploring the genetic basis of human population differences in DNA methylation and their causal impact on immune gene regulation

Lucas T. Husquin, Maxime Rotival, Maud Fagny, Hélène Quach, Nora Zidane, Lisa M. McEwen, Julia L. MacIsaac, Michael S Kobor, Hugues Aschard, Etienne Patin, Lluis Quintana-Murci

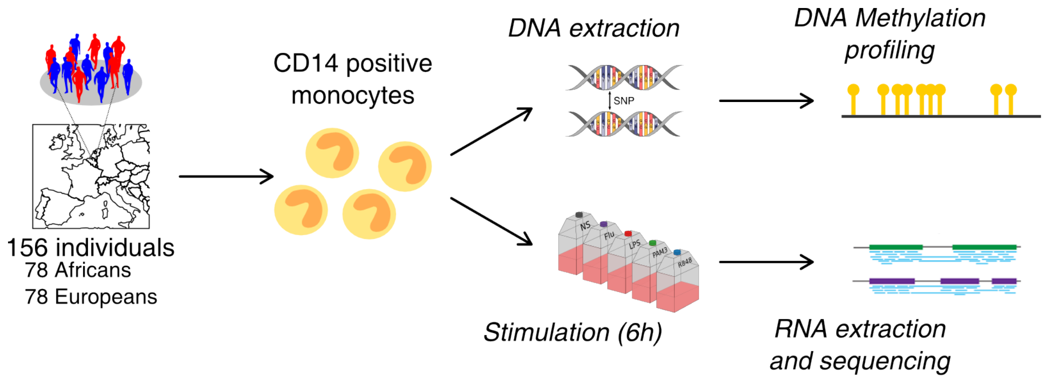

Figure S1 Overview of the EvoImmunoPop experimental setting. DNA methylation profiles and transcriptional responses to various immune stimulations, of primary monocytes from 156 healthy donors of European and African descent.

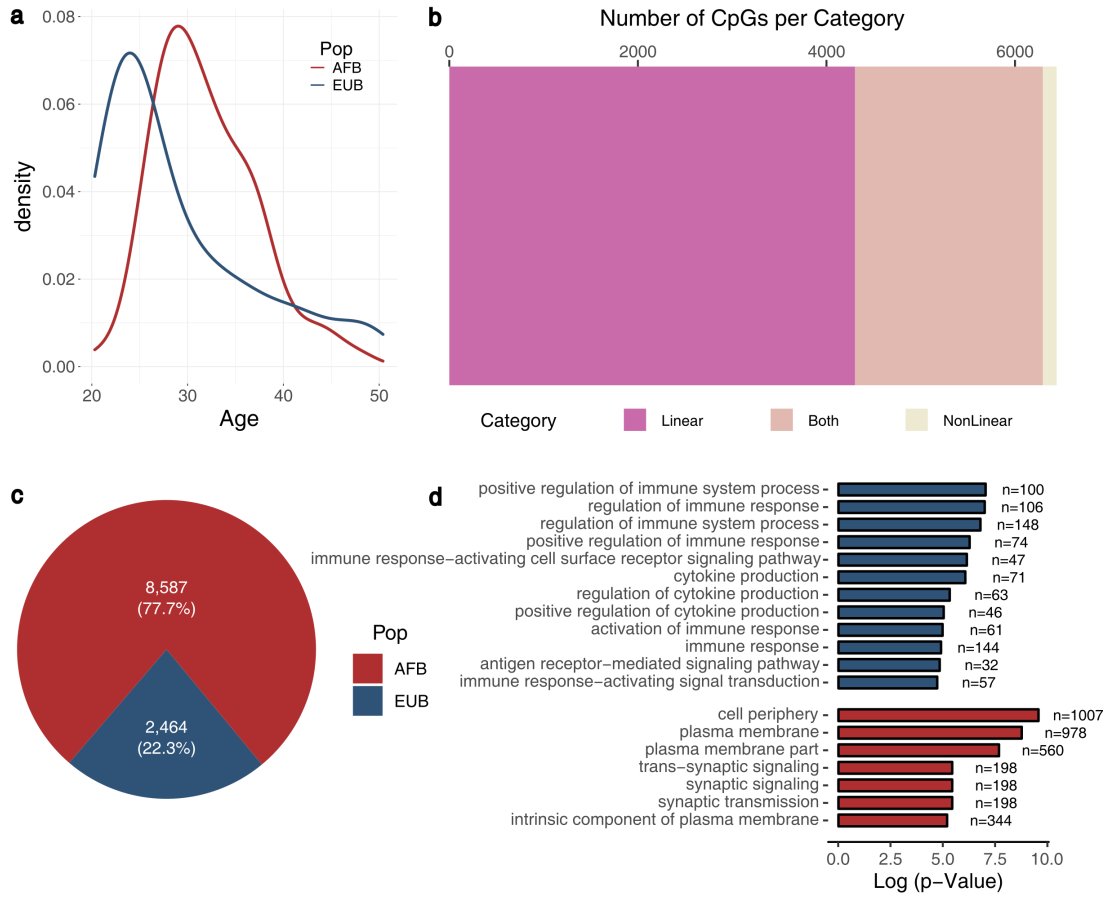

Figure S2 Exploring the non-linear effects of age on DNA methylation. **a** Age density in AFB (red) and EUB (blue) individuals. **b** Number of CpG sites presenting age-dependent methylation changes, as detected by linear regression (magenta), ANOVA (yellow) or both analyses (salmon). **c** Proportion of DMS detected with the ANOVA model that are either hypermethylated in AFB (red) or in EUB (blue) individuals. **d** Gene Ontology (GO) enrichment analyses of AFB- and EUB-DMS. For both groups, the top-GO categories reaching 5% FDR are shown, together with the number of genes per category and the log-transformed FDR-adjusted enrichment *P*-values.

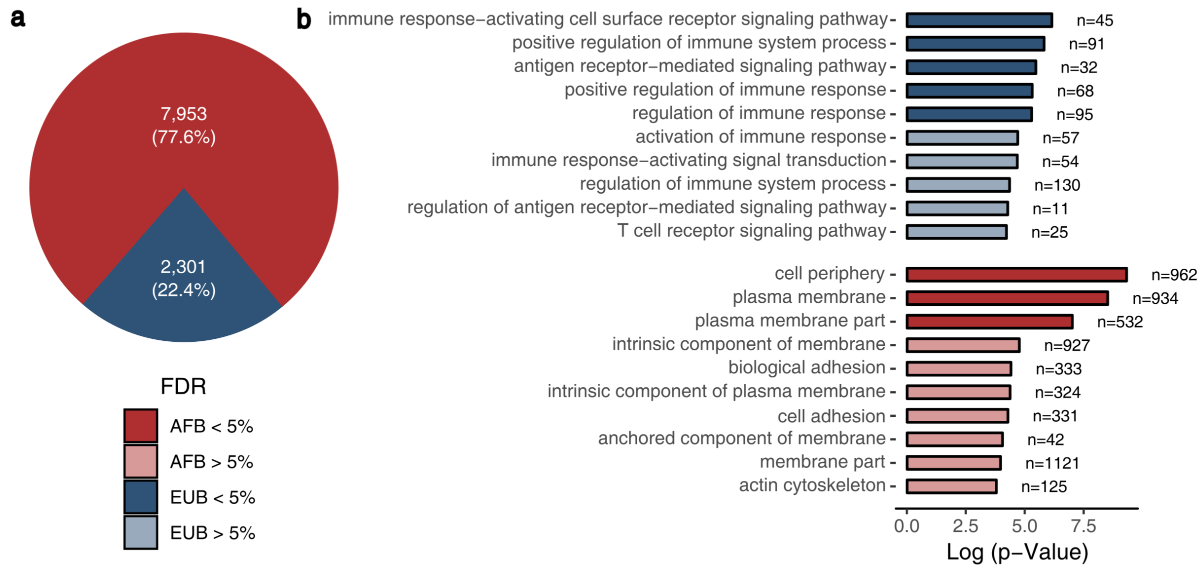

Figure S3 Mono-DMS were detected using the same approach as described in the Methods section, and including the proportions in monocyte subpopulations as covariates. **a** Proportion of Mono-DMS that are either hypermethylated in AFB (red) or in EUB (blue) individuals. **b** Gene Ontology (GO) enrichment analyses of AFB- and EUB-Mono-DMS. For both groups, the ten top-GO categories are shown. Categories reaching 5% FDR are shown in dark red for AFB and dark blue for EUB. The number of genes per category and the log-transformed FDR-adjusted enrichment *P*-values are also indicated.

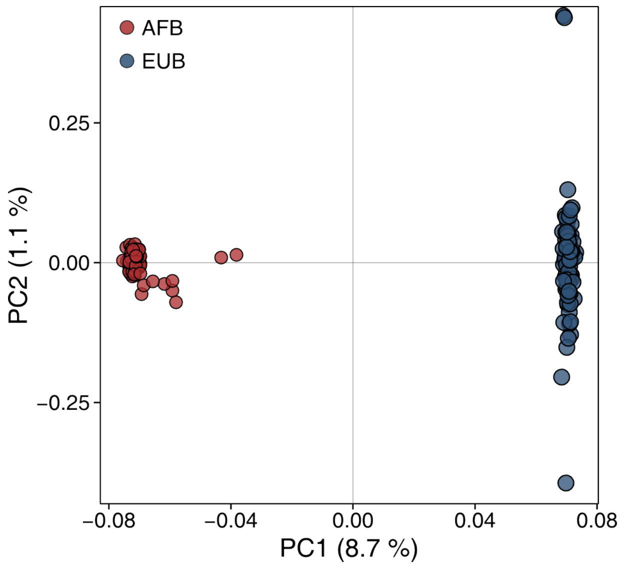

Figure S4 PCA of the genetic data, based on 151,419 SNPs, for Africans (AFB, red dots) and Europeans (EUB, blue dots). The percentages of variance explained by PC1 and PC2 are indicated.

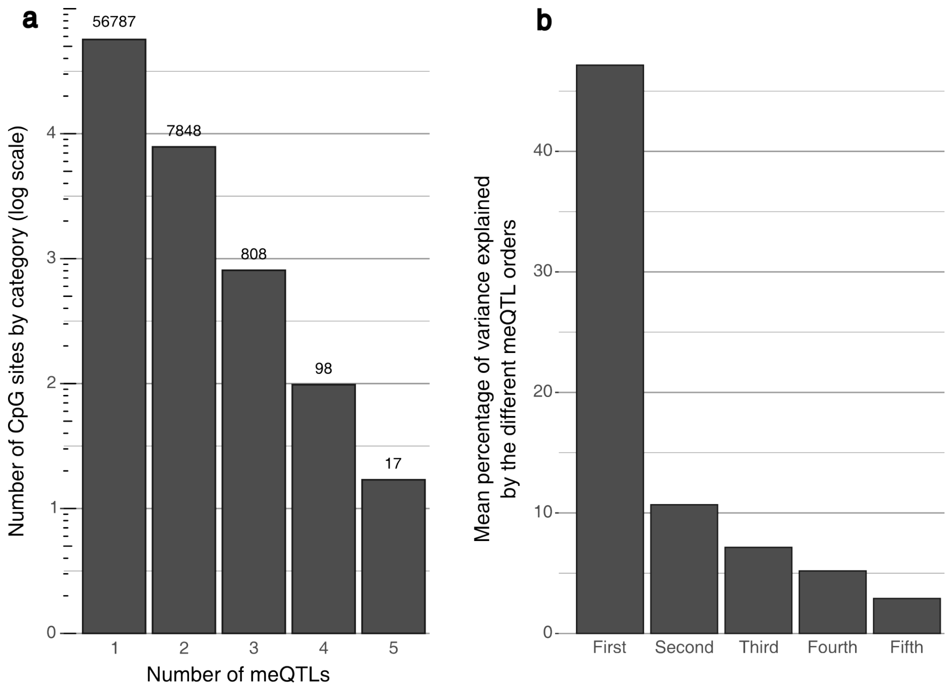

Figure S5 Fine mapping of meQTLs. **a** The number of CpG sites according to the number of associated independent meQTLs is shown on a log-scale. **b** Mean percentage of variance of DNA methylation explained by meQTLs according to the order in which they were detected.

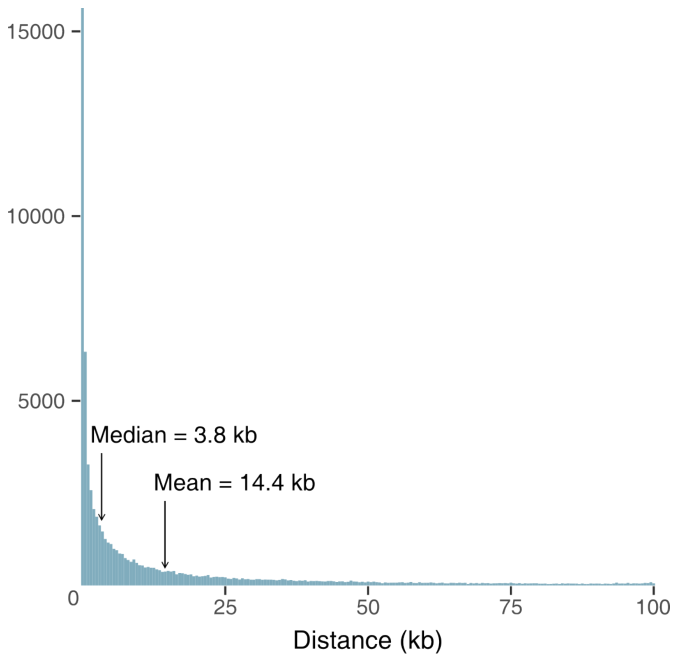

Figure S6 Histogram of physical proximity of *cis*-meQTLs. The distribution of the distances (in kb) between each meQTL and its associated CpG sites is presented, together with the mean and the median value.

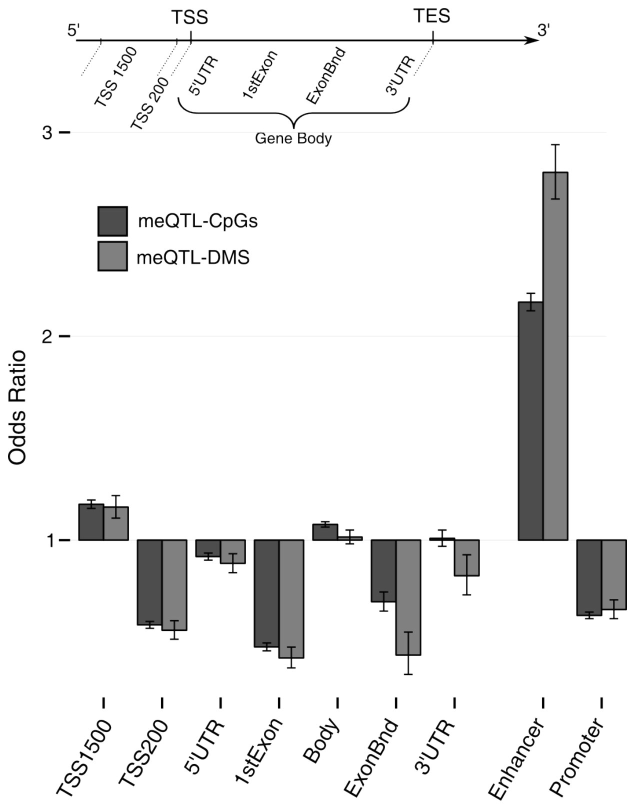

Figure S7 Genomic location of CpG sites associated with a meQTL. meQTL-CpGs are represented in dark grey, and the subset of these CpG sites that were also detected as DMS (meQTL-DMS) in light grey. OR were computed against the general distribution of the 552,141 CpGs of our dataset

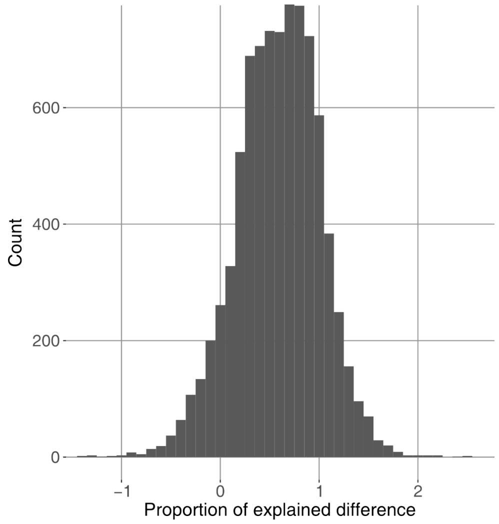

Figure S8 Proportions of population differences in DNA methylation accounted for by genetics. Histogram of the distribution of these proportions, for the 8,459 DMS that were associated with at least one meQTL. Proportions lower than 0 represent situations where genetics has an opposite effect to the observed overall population difference in DNA methylation. Conversely, proportions higher than 1 represent situations where the difference attributable to genetics is higher than that actually observed, indicative of an opposite effect of environmental factors or non-detected independent genetic effects.

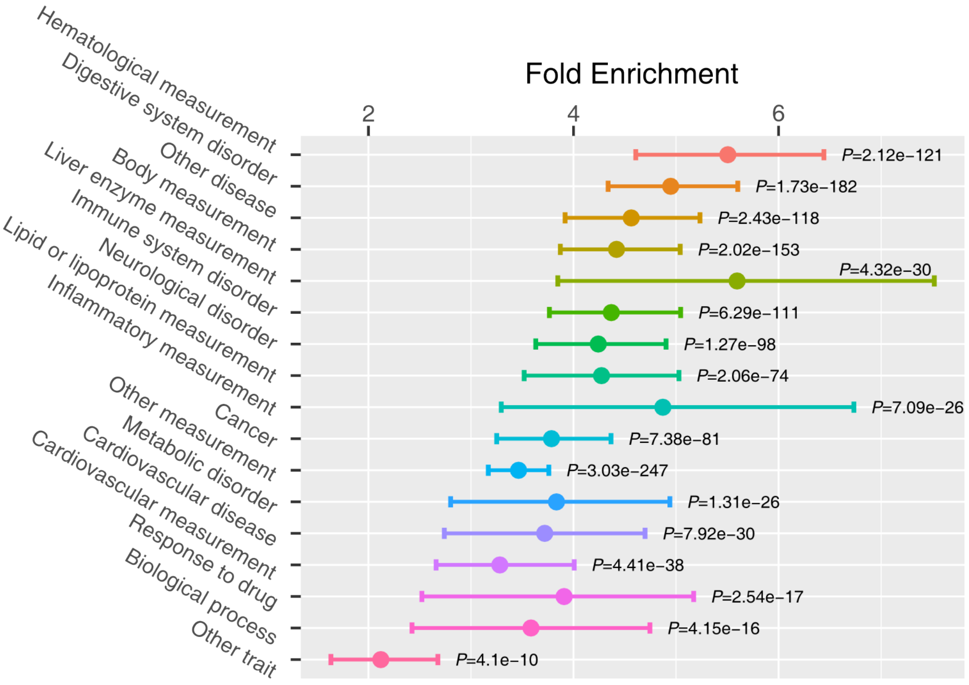

Figure S9 Fold enrichment of meQTLs in GWAS hits. For each of the 17 parental EFO categories, the fold enrichment, the 95% confidence intervals and the associated *P* values are shown.

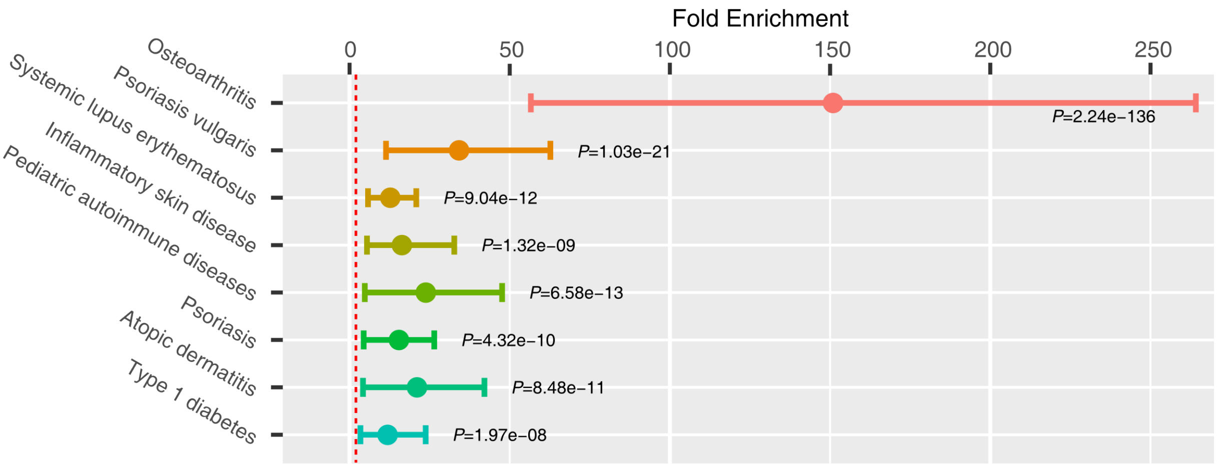

Figure S10 Fold enrichment of meQTLs associated with DMS in GWAS hits related to “immune system disorder”. For the 8 signals that presented the higher lower-bound of confidence intervals, the fold enrichment, the 95% confidence intervals and the associated *P* values are shown.

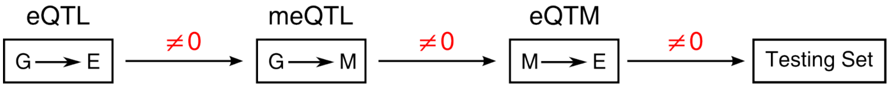

Figure S11 Rationale for the detection of trios to be used for causality inference.

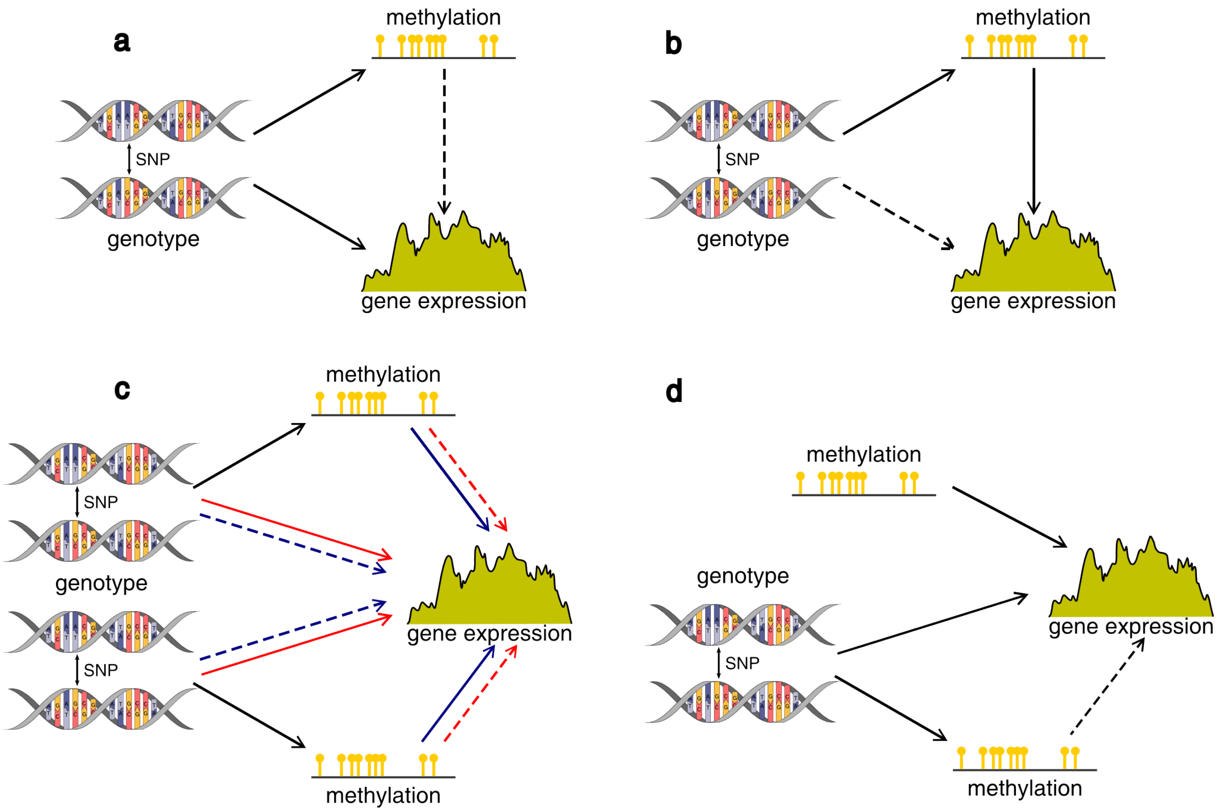

Figure S12 Cartoons of the various simulated scenarios. Plain arrows represent causal relationships, while dashed arrows represent correlations through another relationship. **a-b** Simple situations where either DNA methylation or genetics causally impact gene expression variation. **c** More complex scenarios where gene expression is causally impacted by two independent genetic (red arrows) or epigenetic (blue arrows) variants. **d** Scenario where the CpG site that causally impacts gene expression variation is not under the control of any genetic variant. Note that for all simulated scenarios (**a-d**), similar results between mediation analyses and partial correlations were obtained in terms of sensitivity and specificity (data not shown).

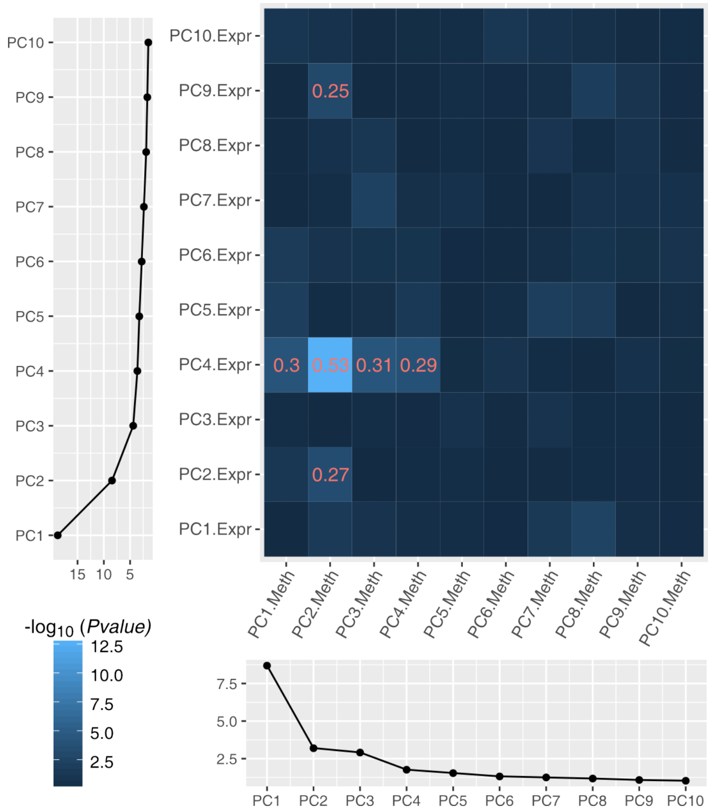

Figure S13 Heat map of correlation between the first ten PCs of expression and DNA methylation. Shades of blue are proportional with the –log10 of the correlation *P* values. In red are given the R^2 of the correlation for cases were $P < 0.001$. Bottom and left panels show the percentage of variance explained by the first ten PCs of gene expression and DNA methylation, respectively.

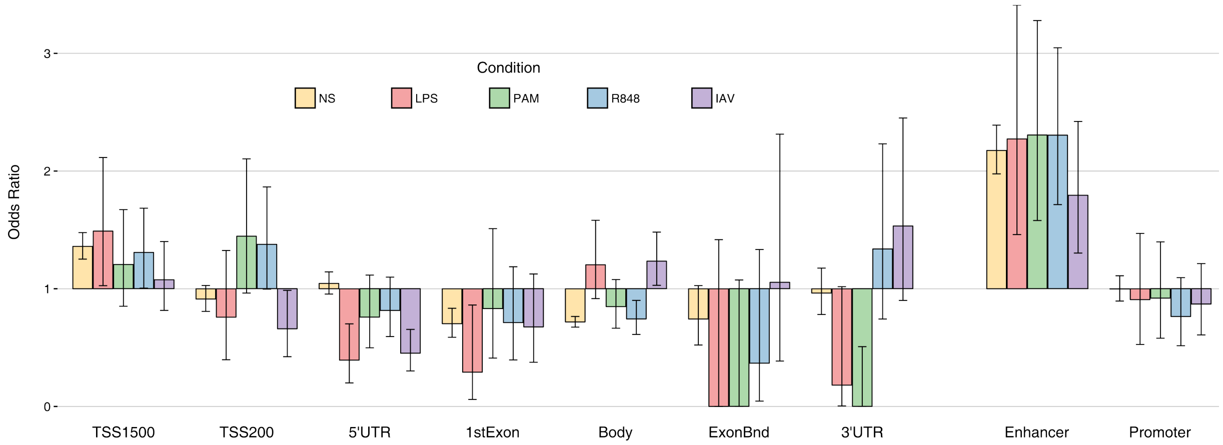

Figure S14 Genomic location of eQTMs (NS) and reQTMs (for all stimulated conditions). Odds ratio were computed against the general distribution of 552,141 CpGs of our dataset.

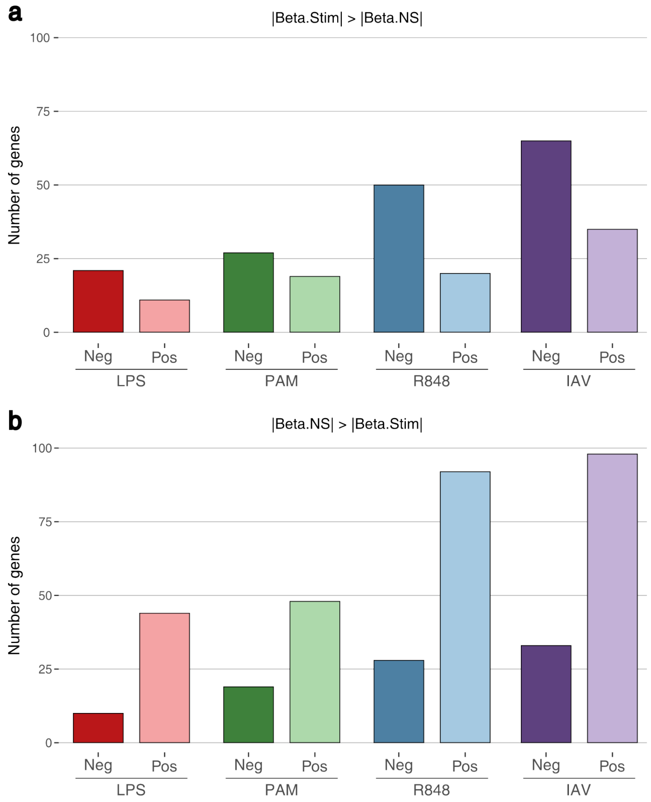

Figure S15 Number of reQTM-genes, per condition, according to the direction of their association with DNA methylation. **a** Cases presenting a stronger expression-methylation association upon stimulation than at the non-stimulated state, **b** Cases presenting a stronger expression-methylation association at the non-stimulated state than upon stimulation.

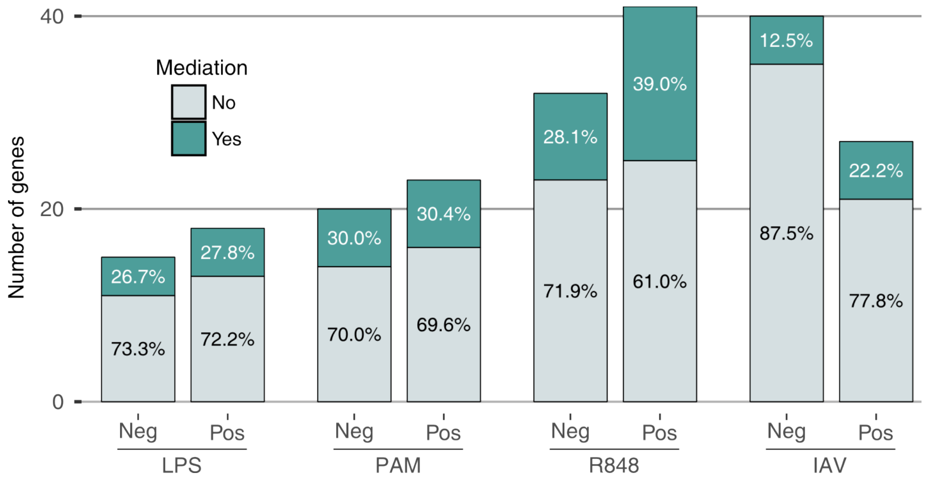

Figure S16 Causality inference upon immune stimulation. Number of mediated and non-mediated reQTM-genes for negative (Neg) and positive (Pos) associations between DNA methylation and fold-changes in expression upon different stimulation conditions. The percentages among these two categories are also indicated.
