# Supplementary material for: Exploring the genetic basis of human population differences in DNA methylation and their causal impact on immune gene regulation
Source: Genome Biol. 2018 Dec 18;19:222. doi: 10.1186/s13059-018-1601-3 (PMC6299574; doi:10.1186/s13059-018-1601-3)
Supplement: Supplementary file 2 — Notes 1–2. (PDF 94 kb) [file 13059_2018_1601_MOESM2_ESM.pdf]

Note 1

Because the age distributions of African- and European-ancestry individuals significantly differ (Wilcoxon P-value = 10^{-4}; **Additional file 1: Fig. S2a**), we investigated the extent to which DNA methylation is non-linearly affected by age in our dataset.We first created a factor variable − grouping individuals by ranges of age (20-25, 25-30, 30-35, 35-40, 40-45, 45-50) − and subsequently applied an ANOVA regression of DNA methylation for each CpG site on this age factor variable, using the *Anova* function from the R package *car*.

$$Anova(lm(Meth \sim AgeClass + Pop)) \qquad (i)$$

Concurrently, we aimed at identifying a linear effect of age on DNA methylation, using a linear model.

$$lm(Meth \sim Age + Pop) \qquad (ii)$$

After correction for multiple testing using the *p.adjust* function from R, with method="fdr", we identified 6,295 and 2,137 CpGs sites that were detected by the linear and non-linear models, respectively. Interestingly, we found an important overlap between these two models, with 1,991 CpGs that were detected by both analyses (**Additional file 1: Fig. S2b**). This indicates that most of the effects of age on DNA methylation are captured by the linear model, with only 146 CpGs for which age has a strict non-linear effect. On the other hand, using a non-linear model appears to considerably reduce power, with 4,158 CpG sites that are found to be associated with age only when using the linear model. To ensure that the population differences in DNA methylation detected were not age-related effects that we failed to adjust for, we repeated our DMS mapping and accounted for putative non-linear effects of age, by using the P-values of the variable *Pop* in model (*i*). After Benjamini–Hochberg correction, we detected 50,353 CpGs (FDR = 1%) that presented a significant difference between AFB and EUB. When restricting this analysis to CpGs that presented a mean difference > 5%, we identified 11,051 DMS. The vast majority of these (11,001 DMS, 99.5%) were detected by our analysis that did not consider the non-linear effects of age. Furthermore, we found the same asymmetry in terms of number of DMS that are hypermethylated in AFB and EUB (**Additional file 1: Fig. S2c**), and similar GO enrichments (**Additional file 1: Fig. S2d**). Of the 1,049 DMS that were not detected by this analysis, with respect to the original 12,050 DMS, only 16 (1.3%) appear to be non-linearly affected by age. This suggests that the difference observed between analyses results from a difference in power, rather than a genuine non-linear effect of age that we failed to adjust for in our original DMS scan.

Note 2

The reverse causation scenario, where the impact of genetic variation on DNA methylation is mediated by gene expression variation, is highly unlikely in our experimental setting (**Additional file 1: Fig. S1**). Given that DNA methylation was obtained from monocytes at *t*=0, while gene expression was obtained at *t*=6h, the reverse causation could only be observed in cases where expression at *t*=6h is a proxy of expression at *t*=0. We nonetheless tested this hypothesis by considering three different models: *Model 1*, independent control of both gene expression and DNA methylation by genetics; *Model 2*, genetic control of DNA methylation mediated by gene expression; and *Model 3*, genetic control of gene expression mediated by DNA methylation. We computed the log-likelihood of these three models:

L(Model 1)= L(M|G) × L(E|G)

L(Model 2)= L(M|E) × L(E|G)

L(Model 3)= L(E|M) × L(M|G)

with G being the genetic variant, M the CpG site, E the gene expression, and L(Y|X) the likelihood of the standard linear model, with Y as the dependent variable and X as the predictor.

We then calculated each model's probability using a uniform distribution of the priors.

$$(1) \qquad P(Model_i|Data) = \frac{P(Model_i)*P(Data|Model_i)}{\sum_i[P(Model_i)*P(Data|Model_i)]}$$

where *P* represents the probability of model i, and $P(Model_1)=P(Model_2)=P(Model_3)=1/3$. The equation (1) can then easily be simplified as:

$$(2) \qquad P(Model_i|Data) = \frac{Likelihood\ (Model_i)}{\sum_i[Likelihood\ (Model_i)]}$$

We calculated the probability of each model for all trios, and assigned each trio to the model presenting the highest probability, which we required to be higher than 0.9. If no models reached such a probability, the trio was declared non-significant. We found that reverse causation was indeed highly unlikely: at the non-stimulated state, only 3.1% of the trios were assigned to *Model 2*, while <1% of the trios were assigned to *Model 2* in the presence of immune stimulation.
